# Concentrated Conditioned Media from Adipose Tissue Derived Mesenchymal Stem Cells Mitigates Visual Deficits and Retinal Inflammation Following Mild Traumatic Brain Injury

**DOI:** 10.3390/ijms19072016

**Published:** 2018-07-11

**Authors:** Kumar Abhiram Jha, Mickey Pentecost, Raji Lenin, Lada Klaic, Sally L. Elshaer, Jordy Gentry, John M. Russell, Alex Beland, Anton Reiner, Veronique Jotterand, Nicolas Sohl, Rajashekhar Gangaraju

**Affiliations:** 1 Department of Ophthalmology, College of Medicine, University of Tennessee Health Science Center, Memphis, TN 38163, USA; kjha@uthsc.edu (K.A.J.); rrajeshl@uthsc.edu (R.L.); slelshaer@gmail.com (S.L.E.); jgentr21@uthsc.edu (J.G.); jrusse74@uthsc.edu (J.M.R.); areiner@uthsc.edu (A.R.); 2 Cell Care Therapeutics, Inc., Monrovia, CA 91016, USA; mickey.pentecost@gmail.com (M.P.); lklaic@cell-care.com (L.K.); abeland16@students.kgi.edu (A.B.); vjotterand@cell-care.com (V.J.); nsohl@cell-care.com (N.S.); 3 Department of Anatomy and Neurobiology, College of Medicine, University of Tennessee Health Science Center, Memphis, TN 38163, USA

**Keywords:** TBI, adult stem cells, TSG6, retinal ganglion cells, oxidative stress, TIMP1, microglia, trans-endothelial electrical resistance

## Abstract

Blast concussions are a common injury sustained in military combat today. Inflammation due to microglial polarization can drive the development of visual defects following blast injuries. In this study, we assessed whether anti-inflammatory factors released by the mesenchymal stem cells derived from adipose tissue (adipose stem cells, ASC) can limit retinal tissue damage and improve visual function in a mouse model of visual deficits following mild traumatic brain injury. We show that intravitreal injection of 1 μL of ASC concentrated conditioned medium from cells pre-stimulated with inflammatory cytokines (ASC-CCM) mitigates loss of visual acuity and contrast sensitivity four weeks post blast injury. Moreover, blast mice showed increased retinal expression of genes associated with microglial activation and inflammation by molecular analyses, retinal glial fibrillary acidic protein (GFAP) immunoreactivity, and increased loss of ganglion cells. Interestingly, blast mice that received ASC-CCM improved in all parameters above. In vitro, ASC-CCM not only suppressed microglial activation but also protected against Tumor necrosis alpha (TNFα) induced endothelial permeability as measured by transendothelial electrical resistance. Biochemical and molecular analyses demonstrate TSG-6 is highly expressed in ASC-CCM from cells pre-stimulated with TNFα and IFNγ but not from unstimulated cells. Our findings suggest that ASC-CCM mitigates visual deficits of the blast injury through their anti-inflammatory properties on activated pro-inflammatory microglia and endothelial cells. A regenerative therapy for immediate delivery at the time of injury may provide a practical and cost-effective solution against the traumatic effects of blast injuries to the retina.

## 1. Introduction

The World Health Organization predicts that traumatic brain injury (TBI) will surpass many diseases, including infectious diseases, as the major cause of death and disability by the year 2020 [1]. It is estimated that about 10–20 million individuals are affected annually by TBI, of which 62% come from automobile vehicle accidents, 10% from sports injuries, and about 2–5% are due to war related injuries [1,2]. In the latter group, studies have shown that 56 to 78 percent of all TBI injuries sustained by U.S. troops are blast-related [3]. Improvements in body armor have reduced mortality rates, but areas of the body that remain exposed such as the face (eyes, in particular), head, and neck remain vulnerable to direct injury that can profoundly impact the neurosensory functions of wounded soldiers [4]. In addition, the secondary displacement of brain tissue can cause acceleration–deceleration injury, further exacerbating the primary pathology in blast or vehicular-related accidents. Both blast and acceleration–deceleration concussive insult lead to axonal injury and cell–cell signaling defects, including injury to the optic nerve, and can set in motion subsequent secondary degenerative events including, but not limited to, progressive vision problems resulting in blindness [5].

Inflammation and oxidative stress underlie several neurovascular degenerative diseases, including light induced retinal degeneration [6,7], diabetic retinopathy [8,9], and TBI [10,11]. Activation and proliferation of microglial cells are hallmarks of ongoing neurodegenerative disease with adverse outcome following mild TBI [12,13,14,15,16]. Microglia can exist across a spectrum of at least three functionally distinct activation states: a ramified morphology state under physiological conditions characterized by motile processes that constantly monitor their immediate surrounding, an activated amoeboid shaped M1-state that release pro-inflammatory cytokines, or a healing type M2-state that produces anti-inflammatory proteins known to play a role in clearance of debris [17]. Glial cells in combination with endothelial cells and pericytes are known to function in the protection of retinal barrier integrity. Any loss of such retinal barrier integrity is associated with a cycle of inflammation, vascular damage, and cell death.

A number of preclinical animal studies and clinical studies have attempted to develop therapeutic strategies or to halt the progression of TBI with no success to date [18]. Adult mesenchymal stem cells (MSCs) derived from the stromal vascular fraction of human adipose tissue (ASC, adipose stem/stromal cells) have therapeutic effects in a variety of neurovascular degenerative models [19]. However, poor cell retention and loss of cell viability and function in the hostile pro-inflammatory environment of injured tissue is a challenge for clinical adoption of cell-based regenerative therapies [20]. Isolation and expansion of a well-defined population of MSCs may also be required for effective therapies as intravitreal injections of poorly defined stromal vascular fraction that contain ASCs has caused retinal detachment and visual loss in patients with age-related macular degeneration [21,22]. Mounting evidence suggests that the paracrine factors from ASCs are sufficient to mitigate a number of inflammatory diseases [23,24,25,26]. Of the many paracrine factors ASCs secrete, vascular endothelial growth factor (VEGF), hepatocyte growth factor (HGF), and granulocyte macrophage colony-stimulating factor (GM-CSF) have been shown to promote endothelial cell survival [27] and proliferation [28]. Furthermore, a variety of chemokines and cytokines that play a major role in anti-inflammatory and immunomodulatory actions of ASCs have been characterized not only from their native condition but also under the influence of Transforming growth factor-β1 (TGF-β1) [29], Tumor necrosis alpha (TNFα) [30], Lipopolysaccharide (LPS) [31], and hypoxia [32]. Therefore, conditioned media, as the basis of a cell-free regenerative therapy, would enable critical manufacturing, storage, and clinical efficiencies that overcome major cost and handling barriers typically associated with regenerative medicine products consisting of living cells.

In this study, using a well-established mild TBI mouse model [10,33], we have evaluated whether the intravitreal injection of ASC concentrated conditioned medium (ASC-CCM) can rescue the visual deficits in TBI. Our data not only demonstrate a beneficial effect of intravitreal injection in the visual deficits but also found it to be safe over a period of a month. Our study also demonstrates proteins that are upregulated in the ASC-CCM when ASCs are pre-stimulated with inflammatory cytokines can suppress microglial activation and protect retinal barrier integrity.

## 2. Results

### 2.1. Concentrated Conditioned Medium from Cytokine Primed ASCs (ASC-CCM) Contains Active Anti-Inflammatory Proteins

**Figure 1:**
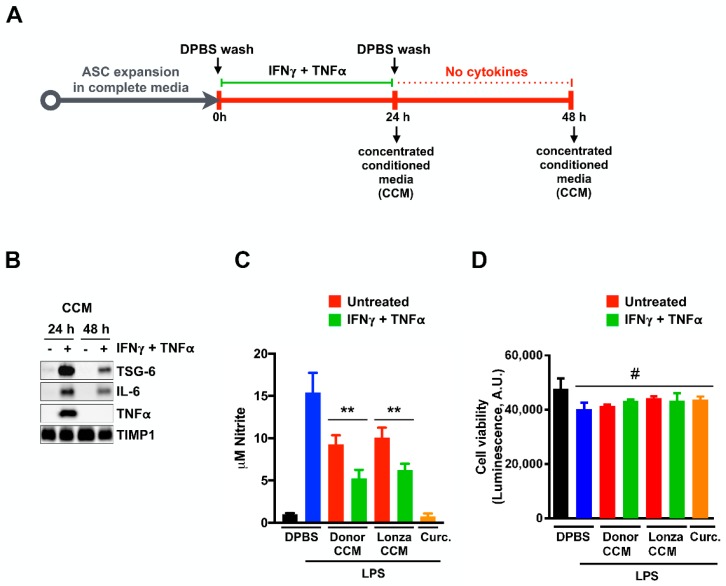
Cytokine priming of adipose stem cells (ASCs) enhances the anti-inflammatory composition of conditioned medium. (**A**) Schema for preparation of exogenous cytokine-stimulated ASC concentrated conditioned medium (ASC-CCM). ASCs are expanded in culture media, washed with Dulbecco’s phosphate-buffered saline (DPBS), and then cultured in media with 10 ng/mL IFNγ, 20 ng/mL Tumor necrosis alpha (TNFα) for 24 h. Cells were then washed with DPBS and cultured for an additional 24 h without IFNγ or TNFα. Conditioned media collected at that time was concentrated (CCM) using centrifugal concentrators with a 3 kDa molecular weight cutoff. (**B**) Immunoblot analysis of TNF-stimulated gene 6 protein (TSG-6), Immunomodulatory Interleukin-6 (IL-6), TNFα, and tissue inhibitor of matrix metalloproteinase 1 (TIMP1) in CCM from untreated or cytokine treated ASCs at t = 24 h or 48 h of the aforementioned preparation scheme. ASC-CCM was normalized to 50 μg/mL in each sample. (**C**) Biochemical assessment of NO release from BV2 cells using the Griess assay. BV2 cells were treated with 5 μg/mL of 48 h CCM from untreated (red bars) or cytokine treated (green bars) donor derived (Donor CCM) or Lonza ASCs (Lonza CCM) for 1 h then with 50 ng/mL Lipopolyaccharide (LPS) for an additional 24 h. To determine baseline and maximum nitrite, BV2 cells were treated with DPBS alone (black bar) or with DPBS and then LPS (blue bar). Curcumin (Curc.; 10 μM) was used as a positive control for suppression of LPS-mediated activation (orange bar). Data represent Mean ± SD from at least three technical replicates. **, *p* < 0.01 (**D**) Luminescence-based assessment of BV2 viability using Cell-TiterGlo. #, *p* > 0.05. Data represent Mean ± SD from at least three replicates.

MSCs can take on an anti-inflammatory and immune-modulatory phenotype when primed with inflammatory cytokines [34]. For example, TNFα and IL-1β are known to induce the expression of the anti-inflammatory protein TNF-stimulated gene 6 protein (TSG-6). We wondered if priming ASCs with cytokines prior to collecting the paracrine factors in the conditioned media could enhance any apparent therapeutic activity (Figure 1A). We first assessed the effect of various inflammatory cytokines on the expression of TSG-6 in ASCs. We confirmed that TNFα or IL-1β individually induce the cellular expression of TSG-6 by ASCs by immunoblot analyses of cell lysates using COX IV as a loading control (Supplementary Figure S1). Surprisingly, a combination of IFNγ and TNFα has a strong synergistic effect on the cellular expression of TSG-6 (Supplementary Figure S1). The relative level of TSG-6 secreted by ASCs into the culture media corresponded to the relative levels in cell lysate, with the greatest abundance in media from IFNγ and TNFα stimulated ASCs (Supplementary Figure S1, Figure 1B). We found that the secretion of tissue inhibitor of matrix metalloproteinase 1 (TIMP1) was largely unaffected by cytokine stimulations and therefore served as a loading control for proteins in the ASC conditioned media (Supplementary Figure S1, Figure 1B).

We next determined whether TSG-6 secretion by ASCs would continue after the removal of the inflammatory cytokines, allowing for the collection of an anti-inflammatory conditioned media. ASCs were cultured until approximately 80% confluence and then treated with media containing IFNγ and TNFα. Following IFNγ and TNFα removal, cells were incubated for an additional 24 h. Conditioned media collected at both the 24 and 48 h time points was concentrated and total protein was measured by Qubit total protein assay (Figure 1A). TSG-6 continued to be secreted into the conditioned media even after IFNγ and TNFα were removed (Figure 1B), albeit at lower amounts. Immunomodulatory Interleukin-6 (IL-6) was also upregulated and secreted into the conditioned media as a result of the pre-stimulation with IFNγ and TNFα (Figure 1B).

It was previously shown that mouse bone marrow MSCs could inhibit the LPS-mediated pro-inflammatory activation of BV2 cells, a murine microglia-like cell line, through TSG-6 [24]. Therefore, we hypothesized that the IFNγ and TNFα primed ASC-CCM might also suppress microglial activation. LPS-activated BV2 cells secrete nitric oxide that decomposes to nitrite, which can be measured from the culture medium using the Griess assay (Figure 1C) and controlled for cell number using a luminescent cell viability assay (Figure 1D). While ASC-CCM from untreated cells could suppress the production of nitrite by LPS treated BV2 cells, IFNγ and TNFα primed ASC-CCM at the same total protein concentration (5 μg/mL) has significantly enhanced activity (*p* < 0.01, Figure 1C). Curcumin, a known anti-inflammatory drug (10 μM), served as a positive control in our assay and DPBS (Dulbecco’s phosphate-buffered saline) as a vehicle control, with and without LPS stimulation of BV2 cells. The suppressive activity of ASC-CCM was not specific to our initial donor cells, as ASC-CCM from a commercial ASC (Lonza) was similarly potent. The IFNγ and TNFα primed ASC-CCM from these commercially purchased cells was used in all subsequent experiments for transferability and generalizability.

### 2.2. ASC-CCM Suppresses LPS and IFNγ Induced Pro-Inflammatory Gene Expression of BV2 Cells

**Figure 2:**
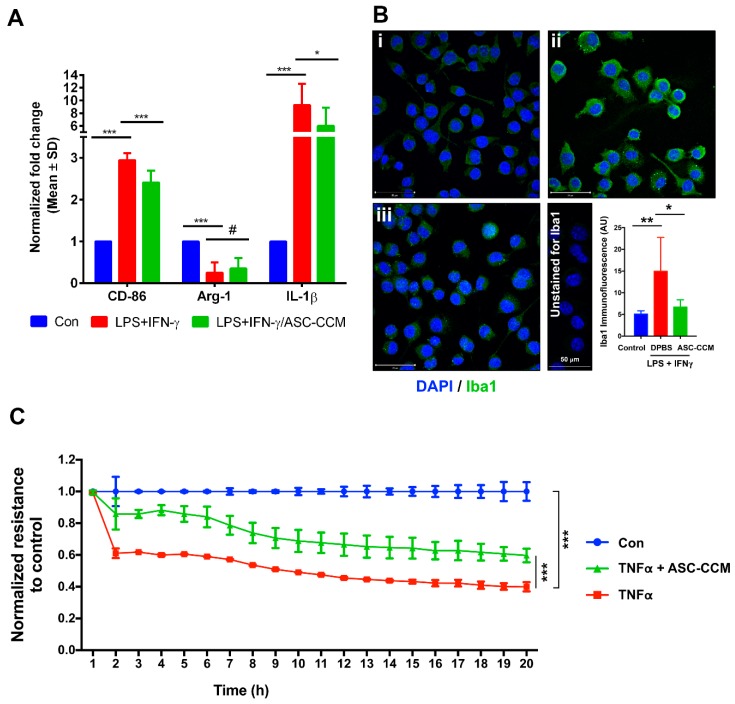
ASC-CCM suppresses microglial activation and improves trans-endothelial resistance. (**A**) ASC-CCM suppresses the LPS (100 ng/mL) and IFNγ (10 ng/mL) induced pro-inflammatory gene expression of BV2 cells. Assessment of gene expression by Sybr Green qPCR and expressed as fold change normalized to internal control (GAPDH) in the study groups. Data represent Mean ± SD from three separate experiments performed in duplicate. *, *p* < 0.05; ***, *p* < 0.001; #, *p* > 0.05. (**B**) ASC-CCM reduces microglial activity as shown by the decreased Iba1 immunoreactivity with LPS and IFNγ stimulated BV2 cells after 12 h exposure. Bar graph shows quantification of mean fluorescence intensity of Iba1. Data are Mean ± SD performed in duplicates. *, *p* < 0.05; **, *p* < 0.01. Scale bar = 50 µm. (**C**) Trans-endothelial resistance is protected by ASC-CCM in vitro. Representative electric cell-substrate impedance-sensing (ECIS) tracings plotted as normalized resistance expressed as Mean ± SD of single experiment performed in replicates with similar data from two independent experiments. ***, *p* < 0.001.

Production and release of cytokines play a central role in the microglia-mediated inflammatory action. The anti-inflammatory capacity of ASC-CCM was evaluated by assessing the expression of IL-1β and CD-86 (early and late markers of the M1 phenotype of microglia) and Arginase-1 (marker of M2 phenotype of microglia) by real-time PCR. Whereas the BV2 cell treated with LPS and IFN-γ significantly increased the gene transcripts of IL-1β (*p* < 0.01) and CD-86 (*p* < 0.01), the expression of Arg-1 decreased (*p* < 0.01) compared to untreated cells. In contrast, cells pre-incubated with ASC-CCM and challenged with LPS and IFNγ significantly reduced the IL-1β (*p* < 0.05), CD-86 (*p* < 0.01) with a trend toward increase in Arg-1 (*p* = 0.25) gene expression (Figure 2A).

### 2.3. ASC-CCM Pre-Treatment Preserves Resting Cell Morphology in LPS and IFN-γ Stimulated BV2 Cells

Alteration of cell morphology is a characteristic feature of microglial activation [35]. We have assessed morphological changes in BV2 cells by inverted phase contrast microscope and F-actin immunofluorescence staining with and without pre-treatment of ASC-CCM. Whereas the morphology of control BV2 cells showed well defined soma with distal arborization, cells treated with LPS and IFNγ demonstrated fewer branches, predominately appearing amoeboid. In contrast, BV2 cells incubated with ASC-CCM preserved the arborization with near complete cell bodies (Supplementary Figure S2). Cells immunostained for F-actin confirmed withdrawn projections from the cell body with LPS and IFNγ stimulation and a complete recovery in cells pre-treated with ASC-CCM that resembled normal untreated cells. Since ionized calcium-binding adapter molecule 1 (Iba1) immunostaining correlates with BV2 microglial activation [36], we assessed Iba1 staining in BV2 cells. Whereas in control group BV2 cells, Iba1 immunoreactivity was minimal, it increased in cells treated with LPS and IFNγ (Figure 2B). On the other hand, cells that were pre-incubated with ASC-CCM, demonstrated a decrease in Iba1 immunoreactivity. The mean total pixel intensity of Iba1 expression in the control group was 1.19 ± 0.17, while the pixel intensity of Iba1 expression in cells treated with LPS/IFNγ was 3.48 ± 1.7 (mean intensity/10,000 μm^2^; *p* < 0.01). Cells pre-treated with ASC-CCM and then treated with LPS/IFNγ showed reduced Iba1 expression (1.56 ± 0.39 mean intensity/10,000 μm^2^; *p* < 0.05; Figure 2B).

### 2.4. ASC-CCM Protects Against TNFα Induced Loss of Endothelial Barrier Integrity

We next tested the ability of ASC-CCM to preserve retinal barrier function in an in vitro model of the retinal endothelial barrier by measuring trans-endothelial electrical resistance (TER) (Figure 2C). Human retinal endothelial cells (HREC) exposed to TNFα induced a sustained reduction in barrier integrity as evidenced by decreased TER at 20 h time point (TNFα, 0.4 ± 0.03; control, 1.0 ± 0.0, *p* < 0.001). On the other hand, these effects were partially rescued by treatment with ASC-CCM (0.59 ± 0.04, *p* < 0.001).

### 2.5. ASC-CCM Suppresses Visual Deficits in Blast Induced Damage

**Figure 3:**
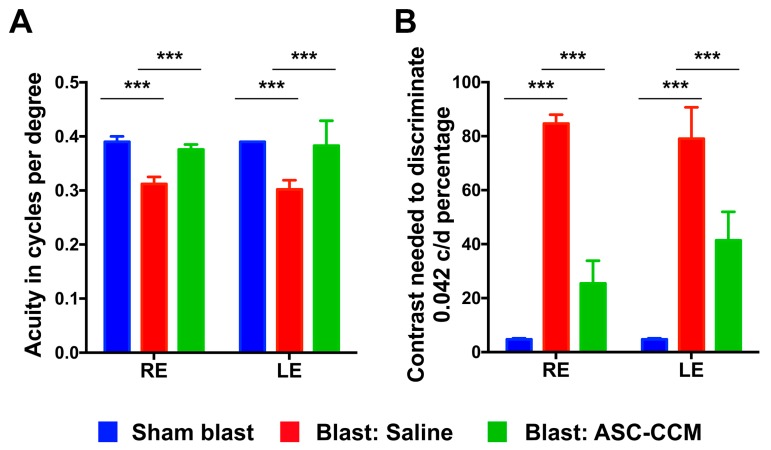
ASC-CCM improves visual acuity and contrast sensitivity in blast injury mice. (**A**) Visual acuity was measured by presenting black and white bars of varying spatial frequencies at 100% contrast (**B**) Contrast sensitivity was measured by changing the contrast gradient that generates tracking at a fixed spatial frequency of 0.042 c/d. Contrast sensitivity in mice is expressed as a percentage, with a higher percent contrast requirement indicating less contrast sensitivity. Data represent Mean ± SEM from *n* = 8–10 animals/group. ***, *p* < 0.001.

Previously using a single focal blast of 50-psi to the left cranium, a consistent visual deficit was reported in both eyes [10]. To better understand the therapeutic benefits of ASC-CCM in blast induced visual deficits, optokinetic measurements were performed four weeks after blast injury in all treatment groups. We assessed visual acuity (spatial frequency threshold) at high contrast (100% contrast) (Figure 3A) and contrast sensitivity at a low spatial frequency threshold [0.042 cycles per degree (c/d), i.e., wide stripes] (Figure 3B). Sham blast mice had a visual acuity of 0.39 ± 0.0 c/d in the left eye and 0.39 ± 0.01 c/d in the right eye. This is within the reported normal visual acuity range for C57Bl/6 mice using the same system [37]. On the other hand, the visual acuity in blast mice was significantly decreased when compared with age-matched sham mice in both the left eye (0.302 ± 0.02; Sham, 0.39 ± 0.01 c/d, *p* < 0.001) and the right eye (0.312 ± 0.01 c/d; Sham, 0.39 ± 0.01 c/d, *p* < 0.001). Interestingly, mice that received ASC-CCM demonstrated a significant improvement in both eyes (left eye, 0.383 ± 0.007 c/d; right eye, 0.376 ± 0.009 c/d, *p* < 0.001). Similarly, the contrast sensitivity of blast mice showed an increase in the contrast needed to detect 0.042 c/d in the left eye (blast, 79.09% ± 11.62%; sham, 4.73% ± 0.41%, *p* < 0.001) and the right eye (blast, 84.67% ± 3.29%; sham, 4.76% ± 0.38%, *p* < 0.001), with a significant improvement observed in mice receiving ASC-CCM for both the left eye (ASC-CCM, 41.41% ± 10.58%, *p* < 0.001) and the right eye (ASC-CCM, 42.43% ± 15.66%, *p* < 0.001). Considering the fact that our single focal cranial blast model caused similar deficits in both eyes and intravitreal injections with ASC-CCM demonstrated similar benefit for both eyes, in subsequent morphological assessments described below we analyzed only left eyes.

### 2.6. ASC-CCM Ameliorates Morphological Changes in the Retina after Blast Injury

**Figure 4:**
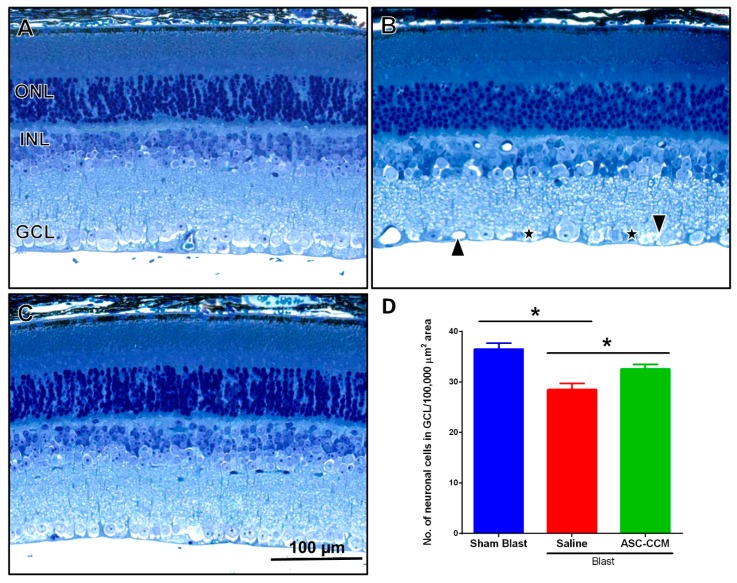
ASC-CCM reduces morphological changes in the retina in blast injury mice. (**A**) Toluidine blue stained sections of retina from the sham group. (**B**) Focal loss of neuronal cells (star) and many vacuolated spaces (arrowheads) in the ganglion cell layer (GCL) were prominent in the blast group. (**C**) Improved morphological appearance observed with ASC-CCM. (**D**) Quantification of mean number of neuronal cells/100,000 µm^2^ area. Data represent Mean ± SEM from *n* = 3 animals/group. *, *p* < 0.05. Scale bar = 100 µm.

In sham group retina receiving saline, the outer nuclear layer (ONL) of central retina contained 10–12 rows of nuclei with lightly dispersed chromatin and intact photoreceptor outer segments, and a well-organized inner nuclear layer (INL) and ganglion cell layer (GCL) (Figure 4A). In blast group retinas receiving saline, the ONL and INL appeared normal when compared to sham group retina. However, focal changes were observed in the GCL, with neuronal cells reduced in blast group retina (Figure 4B). On the contrary, in ASC-CCM injected group retinas after blast, the GCL integrity was maintained as compared to that in blast injury animals receiving saline (Figure 4C). Quantitative analysis utilizing toluidine blue stained light micrographs revealed significant reduction in the neuronal cell count in GCL from blast group mice receiving saline when compared to sham mice (28.49 ± 2.9 vs. 36.49 ± 2.67 cells/100,000 µm^2^ area, *p* < 0.05) with a preservation by ASC-CCM injection as compared to blast mice receiving saline (32.56 ± 2.2 vs. 28.49 ± 2.9 cells/100,000 µm^2^ area, *p* < 0.05) (Figure 4D).

### 2.7. ASC-CCM Reduces Blast-Induced Expression of Glial Fibrillary Acidic Protein (GFAP) in Müller Cells

**Figure 5:**
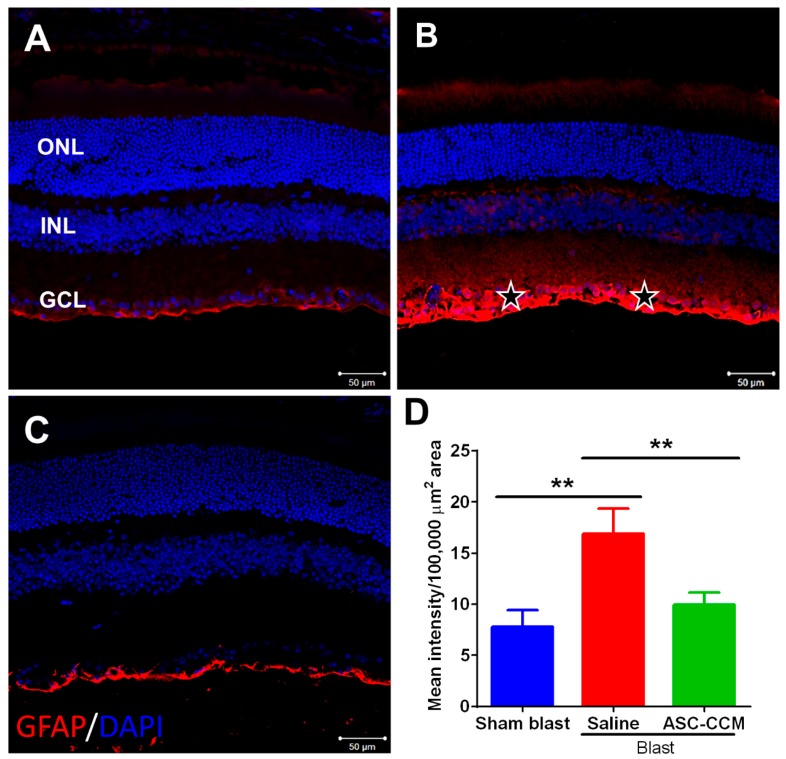
ASC-CCM reduces retinal glial fibrillary acidic protein (GFAP) expression in blast injury mice. (**A**) Normal GFAP immunoreactivity is present in the sham group retina. (**B**) Thirty days after blast exposure, GFAP immunolabeling (red) increases in the nerve fiber layer (NFL, stars) and inner retina. (**C**) Decreased GFAP expression in NFL and inner retina in blast mice with ASC-CCM compared to blast mice with saline. (**D**) Image J quantification of GFAP intensity. Data represent Mean ± SEM from *n* = 6–7 animals/group. **, *p* < 0.01. Scale bar = 50 µm.

Müller glial cells are the principal glial cells of the retina and are found throughout the entire topographic extent of the retina. In response to retinal damage by injury or stress, GFAP expression is upregulated in Müller cells, suggesting reactive gliosis [38]. We determined whether treatment with ASC-CCM in mice subjected to blast injury attenuated Müller glial cell activation (Figure 5). In the sham group, GFAP expression was observed mainly in the nerve fiber layer, NFL (Figure 5A) while in blast group with saline, GFAP expression was found in the Muller glial processes that extended into inner retina (Figure 5B). In contrast, the blast group that received ASC-CCM demonstrated lower levels of GFAP expression (Figure 5C). The mean total pixel intensity of GFAP expression measured from NFL to retinal pigment epithelium in normal sham group retina was 7.7 ± 1.6 while the blast group with saline was 16.9 ± 2.45 (mean intensity/100,000 μm^2^ area; *p* < 0.01, *n* = 7; Figure 5D). On the other hand, blast mice with ASC-CCM showed reduced GFAP expression (9.96 ± 1.17 mean intensity/100,000 μm^2^ area; *p* < 0.01, *n* = 7).

### 2.8. ASC-CCM Reduces Blast-Induced Alteration of Microglia in Blast Injury Mice

The morphology of microglia in response to blast exposure and with ASC-CCM was assessed by Iba1 immunostaining. We and others have shown that Iba1 positive cells are increased in the retina after blast exposure [10,11]. In sham animals, Iba1 immunoreactivity was observed in the INL and outer plexiform layer (OPL) with small cell bodies, thin and regular ramified processes (Supplementary Figure S3A,D,E). On the other hand, in blast exposed retina, thicker processes and swollen cell bodies were observed in the OPL and INL, resembling the amoeboid shape characteristic of activated microglia (Supplementary Figure S3B,F,G). In contrast, in the blast group that received ASC-CCM, near-normal, thin and ramified microglia were seen that possessed the morphology observed in the sham group retina (Supplementary Figure S3C,H,I).

### 2.9. ASC-CCM Suppresses Pro-Inflammatory Gene Transcripts in Blast Mice Retina

**Figure 6:**
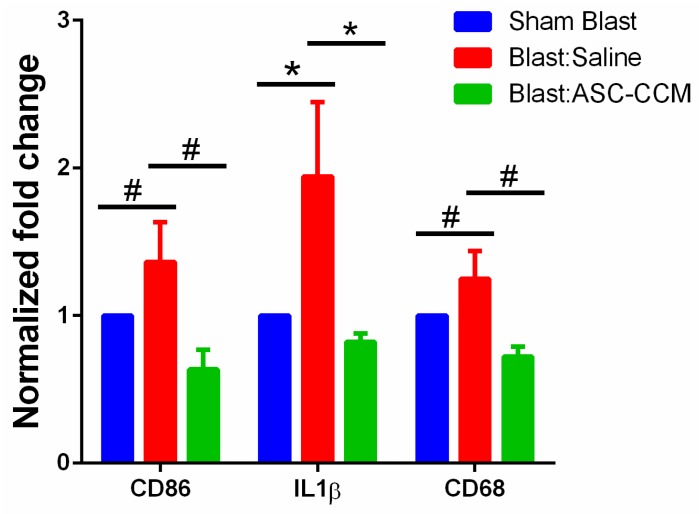
ASC-CCM reduces retinal inflammation in blast injury mice. ASC-CCM reduces retinal inflammation in blast injury mice. Assessment of gene expression by TaqMan qPCR and expressed as fold change normalized to internal control (18s rRNA) in the study groups. Data represent Mean ± SEM from *n* = 3–4 animals/group performed in duplicates repeated two additional times with similar data. #, *p* > 0.05; *, *p* < 0.05.

The anti-inflammatory capacity of ASC-CCM was evaluated by assessing the expression of IL-1β and CD86 (early and late markers of the M1 phenotype of microglia) and CD68 (markers of inflammation) by real-time PCR four weeks after blast injury. Retinal extracts from blast mice receiving saline had significantly (*p* < 0.05) increased abundance of IL-1β gene transcripts compared to sham mice, reflecting microglial activation. Though upward regulation of CD86 and CD68 markers was noted in blast injury group, the results did not reach statistical significance (*p* = 0.07). Interestingly, retinal extracts from mice receiving ASC-CCM demonstrated a significant reduction in all three gene transcripts (*p* < 0.05) compared to blast injury mice receiving saline (Figure 6). Considering the variation in CD86 and CD68 gene expression at four weeks post blast injury, retinas at day 3 post blast injury were also assessed for gene expression. Our data at day 3 demonstrated a significant upregulation in all these genes in blast mice receiving saline (*p* < 0.001) and a significant downregulation in blast mice with ASC-CCM (*p* < 0.001; Supplementary Figure S4).

## 3. Discussion

To the best of our knowledge, this study provides the first demonstration that intravitreal injection of ASC secretome provides protection against visual deficits in the mild TBI model. More importantly, the secretome of cytokine primed ASCs is superior to that of unprimed ASCs, with benefits that include the downregulation of gliosis and inflammation, hallmark features observed in this model. Although more studies are needed, this and our previous study using Streptozotocin-induced diabetic retinopathy [9] suggest that ASCs could be utilized to treat retinal disease or injury.

MSCs are considered a promising cell type with high potential for a regenerative effect in various diseases [19]. ASCs have gained particular traction as a source of therapeutic MSCs due to their easy isolation from an abundant source. Additionally, ASCs may be particularly suited for the treatment of neurodegenerative diseases including retinal diseases [8,39,40]. Although stem-cell clinics offering ASC therapies have gained popularity in United States, there are few regulated clinical trials registered with the Food and Drug Administration (FDA) exploring the safety and therapeutic efficacy of ASCs. There is legitimate concern that stem cell therapies must be scientifically validated and proven safe before clinical trials attempted [41]. Indeed, case reports describe the loss of vision in stem cell clinic patients who have received intravitreal injections of an adipose tissue stromal vascular fraction containing stem cells among other cells [21,22]. In contrast to poorly refined tissue products marketed as “stem cells,” our study suggests that cell free, stem cell-derived factors can fulfill the therapeutic promise of stem cells to alleviate visual deficits and enable the well-controlled, scaled manufacture of a biologic that will meet FDA requirements for human use.

Currently, the main proposed mechanisms through which ASCs are known to regenerate a degenerated tissue include their capacity to home to sites of injury or by releasing trophic factors [28,42,43,44,45]. Importantly, ASCs provide significant complementary support to tissues by the release of anti-apoptotic, anti-inflammatory factors and growth factors [28], that in turn may promote dual beneficial effects addressing both vascular injury and neurodegeneration [8]. However, the specific role of ASC-released paracrine factors in retinal injury is less well studied. The anti-inflammatory or anti-apoptotic molecules that are released by the injected ASCs have not been identified. Here, we have shown that anti-inflammatory proteins are released which could be further enhanced with cytokine stimulation. Our data corroborates several studies that have identified anti-apoptotic and anti-inflammatory proteins such as TSG-6, STC-1, and others in CM that have been shown to mediate some of the pro-survival effects of MSC [25,26,46,47].

A variety of neuroprotective factors have been shown to be released by MSCs including but not limited to BDNF, GDNF, CNTF, PDGF, VEGF, and neurotrophin-4. Accordingly, a broad spectrum of human diseases of the central nervous system including stroke, Alzheimer’s, Parkinson’s are being treated with MSC therapies in clinical trials [48,49]. Similarly, MSC are shown to provide trophic support for the neuroprotection and axon regeneration in animal models of glaucoma, optic nerve transection, light induced photoreceptor damage, and retinitis pigmentosa [50,51,52,53,54]. Our experiments have shown that anti-inflammatory and neuro-protective factors including TIMP1 and TSG-6 are produced by adipose stem cells. Furthermore, we have shown that TSG-6 is upregulated by the synergy of IFNγ and TNFα. Future studies will focus on detailed-omics level identification and characterization of ASC-CCM components, including bioactive proteins and nucleotides associated with extracellular vesicles that may also have therapeutic value.

Studies in animal models suggest that exposure to shock wave blast initially induces production of free radical-generating enzymes in and around brain capillaries. This in turn increases oxidative stress and results in the loss of tight junction proteins, leading to the loss of barrier function and its associated components, including pericytes and astrocyte end-feet [55,56]. We have recently shown in a diabetic rat model, that intravitreal injection of ASC results in a significant decrease in vascular leakage, which we attributed to the fact that ASC are analogous to pericytes and impart natural protection to vasculature [9]. However, it is not known if ASC-conditioned medium, independent of the cells, can protect capillary endothelial cells in the blast injury environment. To this end, we have shown that pre-incubation of retinal endothelial cells with ASC-CCM protects against loss of endothelial junction proteins as evidenced by decreased TER in vitro. This observation is consistent with studies showing that MSC derived conditioned medium or extracellular vesicles protect against endothelial hyperpermeability [57,58,59].

Activation and proliferation of microglial cells is a hallmark of ongoing neurodegenerative disease and has been shown to play a role in the adverse outcome following mild TBI [12,13,14,15,16]. Activated microglia exist in a phenotypic spectrum across several activation types with different functions that include pro-inflammatory cytokine producing M1-state or more homeostatic M2 phenotype that produce anti-inflammatory proteins and help in debris clearance [17]. Although one of the major mechanisms of immunomodulation by MSCs is the regulation of T and NK cells, MSC have also been shown to polarize macrophages from the M1 phenotype towards the M2 phenotype [60]. Macrophage polarization has been observed in several animal models including wound healing [61], brain/spinal cord injuries [62], insulin resistance in obese mice [63], and diseases of the heart [64], lung [65], and kidney [66]. Most importantly, intracerebroventricular delivery of bone marrow-MSC in mice with TBI led to upregulation of the M2 phenotype with significant improvement in neurological functions and repair of the lesioned microenvironment [67]. In accordance with these studies, our study with BV2 microglia demonstrated that ASC trophic factors not only suppressed the M1 phenotype in vitro but also normalize microglia in vivo.

While retinal microglial cells appear to play an early role in degenerative changes, reactive astrocytosis and gliosis, indicated by increased GFAP immunoreactivity in astrocytes and Müller glia, are suggested to be later events [68]. We find increased GFAP immunoreactivity in the retina in blast animals in agreement with other studies of TBI models [10,11]. We showed that GFAP immunoreactivity is decreased in animals that received ASC-CCM. Increased distribution of GFAP throughout Muller glia is a common feature of a variety of retinal diseases, and correlates with neuronal degeneration and loss, resulting in retinal thinning, observed in animal models [38]. Indicative of neuronal degeneration, we observed focal loss of neuronal cells in the GCL of blast retina. This pathology is corrected by intravitreal injection of ASC-CCM. Thus, our data suggests that the trophic factors from ASCs may rescue ganglion or other neuronal cell damage in the blast retina. Since retrograde degeneration of retinal ganglion cells has been linked to head trauma in human subjects [69], ASC-CCM may have multifactorial benefit to the retina.

We recognize that there are other blast injury models to study visual deficits. Direct ocular blast injury by Rex and colleagues [70,71,72] and our TBI model, produce similar functional and neuropathological abnormalities that present themselves along a similar time course. The extent of optic nerve injury appears similar in our TBI model and the direct ocular blast model. A salient difference between the two models is that direct ocular blast lowers IOP, but blast TBI does not. Although beyond the scope of current work, future studies should test the effects of ASC-CCM in direct ocular blast injury. The materials and methods used here can be applied to determine whether ASC-CCM can mitigate direct ocular blast injury since both the ocular blast model and our TBI model use the same blast instrument, albeit at different blast pressure settings (18-psi for ocular blast) and mouse alignment.

In line with a number of experimental ocular cell therapies, we deliver the ASC-CCM close to the damaged retinal vasculature and neurons through intravitreal injection. However, we recognize that while our data suggests intravitreal injections are efficient, the route of injection may not represent the optimal delivery method for human clinical trials. This concept is supported by the marked effects of intravenous injection of ASC paracrine factors that impart neuroprotection in the brain [42]. However, it should also be noted that a study of MSC intravenous infusion into a rodent TBI model did not find it similarly beneficial [73]. This highlights the potential shortcomings of acute, single-dose, intravenous therapy. In addition, the onset of neurodegeneration in TBI may take days to weeks. Therefore, studies are needed to evaluate the relative merit of alternative routes and modes of delivery including continuous infusion.

**Figure 7:**
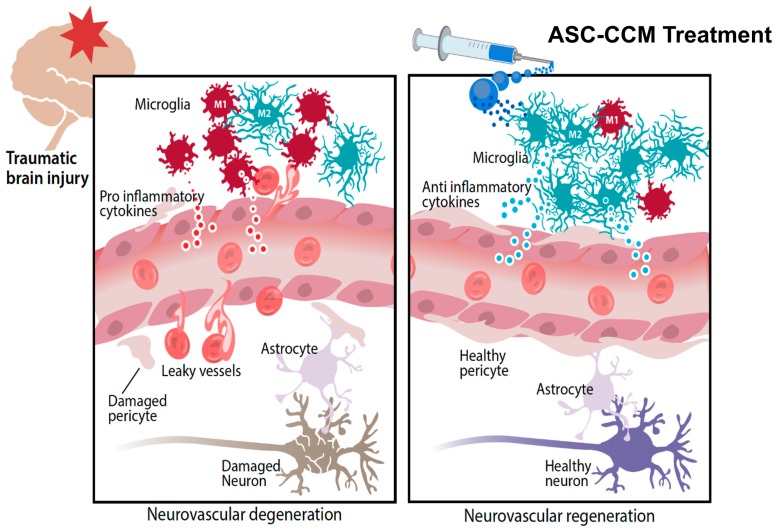
Hypothetical model of ASC-CCM therapy for visual deficits of mild traumatic brain injury (TBI). Activation and proliferation of microglial cells is a hallmark of ongoing neurodegenerative disease and has been shown to play a role in the adverse outcome following mild TBI. Microglia become polarized towards an M1 phenotype after TBI, releasing pro-inflammatory cytokines that result in neurodegeneration and vascular permeability, the hallmarks of visual deficits. On the other hand, treatment with ASC-CCM results in a restored M1-M2 balance leading to expression of anti-inflammatory cytokines and possibly regenerating pericyte–endothelial connections eventually leading to protection against visual deficits.

In conclusion, our findings suggest that ASCs respond to an inflammatory milieu by secreting several therapeutically valuable proteins and exhibit their anti-inflammatory properties on activated pro-inflammatory microglia. We expect similar mechanism or action may be operative in vivo (Figure 7). Our ultimate goal is to manufacture ASC-CCM as a cell-free and battlefield ready regenerative therapy for immediate delivery at the time of injury. This approach will provide a practical and cost-effective solution against the traumatic effects of blast injuries to the retina.

## 4. Materials and Methods

### 4.1. Adipose Derived Stromal Cell (ASC) Culture and Conditioned Medium Preparation

Studies involving human adipose tissue sample collection were approved by the International Cellular Medicine Society, as well as approved as an exempt study by UTHSC Institutional Review Board (16-04861-NHSR, 10/06/2016) and HRPO, US Army Medical Research and Materiel Command, in accordance with relevant guidelines and regulations following the tenets of the Declaration of Helsinki. The adipose specimens were obtained from elective surgical procedures and were deemed normal medical waste products resulting from these procedures. Informed consents were obtained, however, only de-identified specimens were sent to the laboratory for further processing. All donors underwent blood testing at the time of the procedure and no blood-borne pathogens were identified. Human subcutaneous adipose tissue samples obtained from lipoaspiration procedures were processed to isolate ASCs as described by us previously [9,74]. Commercial ASCs were purchased from Lonza (Walkersville, MD, USA). To prepare ASC concentrated conditioned medium, cells were seeded at a density of 5 × 10^3^ cells/cm^2^ in MEM-alpha with 10% FBS. Media was changed at day 3 post seeding. At day 5 post seeding, cells were washed twice with DPBS and then primed with TNFα (R&D Systems, Minneapolis, MN, USA) and INFγ (R&D Systems) in basal MEM-alpha for 24 h. Cells were then washed twice with Ca- and Mg-free DPBS and incubated with fresh medium for additional 24 h. The conditioned medium was then collected, centrifuged to remove cellular debris, and the supernatant was concentrated using an Amicon Ultra centrifugal filter (MilliporeSigma, Burlington, MA, USA). For in vivo experiments 20× concentrated conditioned media was used.

### 4.2. Microglial Cell Culture and Activation

The mouse microglial cell line, BV2, was a kind gift from Professor Grace Sun, PhD, University of Missouri, Columbia, MO, USA. BV2 cells were routinely grown in 100 mm cell culture dishes in Dulbecco’s Modified Eagle Medium (DMEM) with 10% fetal bovine serum and antibiotics. For activation experiments, cells were detached using TrypLE (Thermo Fisher Scientific, Waltham, MA, USA) followed by gentle scrapping and counted, seeded at 5 × 10^5^ cells/cm^2^ of a 24-well plates or 1 × 10^5^ cells/cm^2^ for a 96-well plate. After cells attached briefly for about 4 h, they were stimulated with LPS (100 ng/mL) and IFNγ (10 ng/mL) for 12 h. In some experiments, cells were pre-incubated with ASC-CCM for 6 h before stimulating them with LPS/IFNγ.

### 4.3. Nitric Oxide Release Assay

BV2 cells maintained in DMEM 5% FBS 1% Corning™ Antibiotic-Antimycotic solution were washed once in DPBS then harvested gently with a lifter blade cell scraper into an assay medium and seeded into 96-well plate. Cells were incubated in a humidified 5% CO_2_ incubator at 37 °C for 1–2 h then treated with 10 µL ASC-CCM, DPBS as a vehicle control, or 10 μM curcumin as a positive control for 1 h, before addition of LPS, (50 ng/mL). After 24 h of incubation in a humidified 5% CO_2_ incubator at 37 °C, nitrite concentration in media was determined using the Greiss Reagent System according to the manufacturer’s instructions (Promega, Madison, WI, USA). Cell viability post-treatment was determined using CellTiter-Glo^®^ Assay according to the manufacturer’s instructions (Promega).

### 4.4. Gene Expression Analysis

Total RNA was isolated from 1 × 10^5^ BV2 cells or from individual mouse retinal extracts immediately after euthanasia using NucleoSpin^®^ RNA Plus kit (Macherey-Nagel GmbH (available online: http://www.mn-net.com/)), following the manufacturers protocol. Subsequently, the resulting 75 ng mRNA sample served as a template for real time qPCR using TaqMan probes for animal tissue RNA or the Sybr Green method for BV2 cell RNA (Table 1) and accompanying Master Mix (Applied Biosystems, Foster City, CA, USA). PCR amplification was carried out using Quantstudio3 (Applied Biosystems) with cycle conditions (initial cycle: 50 °C for 5 min, initial denaturation 95 °C for 20 s, 40 cycles of denaturation 95 °C for 3 s, and annealing/extension of 60 °C for 30 s). The expression levels of gene transcripts were determined using 2^−ΔΔ*C*t^ and normalized to internal control.

### 4.5. Western Blot Analysis

ASC-CCM samples were normalized by total protein using a Qubit Protein Assay Kit and a Qubit fluorimeter, subjected to SDS-PAGE under reducing conditions and transferred to immobilon-FL PVDF membrane (MilliporeSigma). For primary detection, goat polyclonal and mouse monoclonal anti-TSG-6 were used at 1:2500–1:5000 (R&D systems), rabbit monoclonal anti-COX IV was used at 1:5000 (LI-COR, Lincoln, NE, USA), goat anti-IL-6 was used at 1:500 (R&D systems), goat anti-TNFα was used at 1:1000 (R&D systems), and rabbit polyclonal anti-TIMP-1 was used at 1:5000. IRDye 680RD and IRDye 800CW conjugated anti-IgG secondary antibodies of appropriate species reactivity were used at 1:5000 for secondary detection. Immunoblots were imaged on a LI-COR Odyssey infrared imager according to the manufacturer’s instructions (LI-COR).

### 4.6. Immunocytochemistry

Immunocytochemistry was performed to reveal localization of Iba1 and F-actin in the BV2 cells. 2 × 10^5^ BV2 cells were grown on 10 mm round coverslips placed in a 24-well plate. After 12 h of experiment with different conditions, cells were fixed with freshly prepared 4% paraformaldehyde for 20 min, blocked with 5% normal horse serum, 1% bovine serum albumin, 0.01% Triton-X for 1 h at room temperature. This was followed by staining for Iba1 (dilutions: 1–2 µg/mL, mouse monoclonal, catalog number: 019-19721, Wako, Richmond, VA, USA) and F-actin (dilutions: 1–2 µg/mL, mouse monoclonal, catalog number: ab205, Abcam, Cambridge, MA, USA) for 24 h at 4 °C. Thereafter, cells were washed with 0.1 M PBS containing 0.01% Triton-X and incubated with secondary antibody (goat anti-mouse Alexa Fluor 488 for Iba1 and goat anti-mouse Alexa Fluor 546 for F-actin, both Thermo Fisher Scientific, 1:200). To identify cells, their nuclei were stained by incubating with DAPI for 2 min. Cells without exposure to the primary antibody were used as negative controls for immunostaining. Stained cells on coverslips were mounted using aqueous mounting medium (Lab Vision PermaFlour, Thermo Fisher Scientific) and were visualized using a Zeiss LSM 710 laser scanning confocal microscope with a 40× oil immersion objective. Quantification of pixel intensities (relative fluorescence units) for Iba1 was analyzed from at least 5 images from different locations per well and expressed as an average of the experimental groups.

### 4.7. Retinal Endothelial Cell Permeability In Vitro

Measurements of trans-endothelial electrical resistance (TER) were performed according to our previously published method that utilizes the measurement of electric cell-substrate impedance-sensing (ECIS; Applied Biophysics, Troy, NY, USA) [75]. Human retinal endothelial cells (HREC; Cell Systems, Inc., Kirkland, WA, USA) were seeded at a density of 5 × 10^5^ cells/mL on gold electrodes (8W10E+; Applied Biophysics, Inc.) and grown for 16 h until maximum resistance was attained (~1200 Ω). Cells were treated with TNFα (1 ng/mL; Sino Biological Inc., Wayne, NJ, USA) with and without ASC-CCM (20 μL/well), and changes in resistance were monitored for up to 18 h. Resistance values for multiple wells, at 4000 Hz, were normalized to an identical starting resistance value and averaged and presented as normalized resistance over time.

### 4.8. Animals and Study Groups

Animal studies were approved by the Institutional Animal Care and Use Committee, UTHSC, Memphis (IACUC ID: 16-110) and USAMRMC Animal Care and Use Review Office (Protocol No. VR150072, 12/05/2016) following the guidelines DOD Instruction 3216.01, “Use of Animals in DOD Programs”. Male adult 12 week old C57Bl/6 mice were purchased from The Jackson Laboratory (Bar Harbor, ME, USA) and kept under controlled temperature (21–23 °C) and lighting conditions (12 h Light/12 h dark cycle). Animals were provided with access to food and water ad libitum. About 24 h prior to blast injury, animals received 32 mg/mL Acetaminophen suspension (Infant’s Tylenol, Cherry flavor, Walgreens Pharmacy) provided in drinking water, yielding a dose of 300 mg/kg/day. After 72 h post blast injury, normal water was provided. All experiments were performed in 2–3 batches with each batch consisting of 8 animals in a sham blast group that received saline, 8 animals in a 50-psi blast group that received saline, and 10 animals in a 50-psi blast group that received ASC-CCM. Experiments described in this study were performed 4 weeks after intravitreal injections; except for studies involving expression of gene transcripts were in addition performed 3 days post injections.

### 4.9. Blast Injury

The TBI mouse model was performed as described previously [10]. Briefly, mice were anesthetized subcutaneously with ketamine (50 mg/kg) and dexmedetomidine (0.25 mg/kg) cocktail and the left side of the cranium was shaved prior to sham or 50-psi blast. Mice were secured within a clear polyvinyl chloride (PVC) pipe with a hole that exposes the parietal region of the left side of the mouse head between the ear and the eye to the blast, while the rest of the mouse is shielded from the blast by the pipe. A foam rubber cushions the mouse to stabilize it and minimize head displacement. A pressure transducer (STJE Sensotec pressure transducer, Honeywell, Morristown, NJ, USA) that is fitted to the gun barrel assessed the pressure output and analyzed using Labview software (National Instruments, Austin, TX, USA). The 0-psi blast animals served as sham blast controls. Within 5 min of the blast procedure, animals received intravitreal injections and reversal from anesthesia using Atipemazole Hydrochloride (0.25 mg/kg). Animals were monitored on a daily basis for 3 days and used for subsequent analysis at 3 days or 4 weeks post blast injury.

### 4.10. Intravitreal Injections

For intravitreal injections mice under ketamine-dexmedetomidine cocktail were placed on a heating pad under a stereo microscope, pupils dilated with 1% tropicamide and 0.5% proparacaine, a local anesthetic applied. Intravitreal injections (1 μL of ASC-CCM or 1 μL of saline) were performed with a 30-gauge microsyringe (Hamilton, Reno, NV, USA), on the temporal side of the eye, 2 mm posterior and parallel to the limbus. Animals were reversed from anesthesia using Atipamezole Hydrochloride and returned to cages.

### 4.11. Optokinetic Reflex Measurements

To assess visual function, optokinetic reflex measurements were made 4 weeks post blast. The untrained, unrestrained awake mice were placed on a platform inside the OptoMotry virtual reality optokinetic reflex system to quantify the visual acuity and contrast sensitivity thresholds (OptoMotry, CerebralMechanics, Lethbridge, AB, Canada) as described previously [37,76]. Visual acuity was assessed at 100% contrast by varying spatial frequency threshold. Contrast sensitivity was assessed in mice by varying the contrast at 0.042 cycles per degree (c/d) of spatial frequency threshold.

### 4.12. Tissue Preparation

Post euthanasia eyes from all groups were enucleated, and lens and vitreous were removed by cutting through cornea. Retinal eyecups were fixed in 4% paraformaldehyde in 0.1 M phosphate buffer (PB) for 4 h at 4 °C. Following this, eyecups were cryopreserved in 15–30% sucrose in 0.1 M PB, embedded in OCT in a cryostat (Microm-HM 550, Thermo scientific) at −20 °C, and sectioned at 12 µm thickness along a dorsal to ventral axis. Sections were placed on to L-poly lysine coated slides and stored at −20 °C for further use.

### 4.13. Immunohistochemistry (IHC)

IHC was performed to localize the expression of target proteins. Cryosections were washed three times with 0.1 M phosphate buffer saline (PBS) and 0.01% Triton-X and immersed in 5% normal serum in 0.1 M PBS for 1 h to block non-specific binding sites. Retinal sections were then incubated in primary antibodies against GFAP (dilutions: 2 µg/mL, rabbit polyclonal, catalog number: ZO334, Dako) or Iba1 (dilutions: 4–5 µg/mL, rabbit polyclonal, catalog number: 019-19741, Wako) for 48 h at 4 °C. After three consecutive washes with 0.1 M PBS-Triton-X, sections were incubated in secondary antibodies (goat anti-rabbit IgG Alexa Fluor 546, dilution: 2 µg/mL, Thermo Fisher Scientific) for 4 h at room temperature. Sections were then washed, incubated with DAPI for nuclear staining and mounted (Lab Vision^TM^ PermaFlour^TM^, Fisher Scientific). Retinal sections were examined under a Zeiss LSM 710 laser scanning confocal microscope with a 20× objective with suitable filters. Tissue sections without exposure to the primary antibody were used as negative controls for immunostaining. Quantification of pixel intensities (relative fluorescence units) of each GFAP-immunolabeled Müller cell was analyzed from at least 3 sections (from NFL to RPE) per eye, 3 areas per retina (two mid peripheral and a central) by an investigator blinded to the groups and expressed as mean intensity per 100,000 um^2^ of the retina.

### 4.14. Evaluation of Retinal Changes by Light Microscopy

After immediate excision of the cornea, lens and vitreous, the remaining eyecups from different groups were fixed in 2% paraformaldehyde and 2.5% glutaraldehyde in 0.1 M PB (pH 7.3) for 4 h at 4 °C. After wash, samples were osmicated for 1 h, dehydrated in acetone, and embedded in Araldite CY212. Thin sections (1 µm) were stained in toluidine blue and montage images of the entire retinal section were captured using Lionheart™ FX Automated Microscope (Biotek US., Winooski, VT, USA) with a 20× objective. The number of neuronal cells were counted in the entire GCL from temporal to nasal along the horizontal meridian from two distinct sections per eye from three different animals per group. Glial cells were excluded by size and their intense basophilic staining. The total number of neuronal cells in the GCL was expressed per 100,000 µm^2^ area of the retina.

### 4.15. Statistical Analysis

Results are expressed as mean ± SD for all in vitro experiments and mean ± SEM for all in vivo experiments. Pairwise *t* tests were run in order to calculate the *p*-values for comparisons between the individual groups. Optokinetic reflex measurements, neuronal counts in the GCL layer, and GFAP expression were compared by ANOVA followed by post hoc *t*-tests with the Bonferroni correction for multiple group comparisons using the bioconductor limma statistical package or GraphPad Prism software. For gene expression analysis, Benjamini Hochberg method was used to calculate the false discovery rate and adjusted *p* values for false discovery corrections due to multiple gene testing. A sample size of three animals was significant given a power analysis using alpha of 0.05, the variance, and standard deviation of the pilot study. A *p*-value < 0.05 was considered to be statistically significant.

## Figures and Tables

**Table 1:** List of Gene transcript TaqMan® Probes\Primers used in the study.

**Genes**	**Taqman Assay ID**	**Reference Sequence**
18S ribosomal RNA (*18s*)	Mm04277571	NR_003278
Interleukin 1 β (*Il1β*)	Mm00434228_m1	NM_008361.3
Cluster of Differentiation 68 (*Cd68*)	Mm03047343_m1	NM_001291058.1
Cluster of Differentiation 86 (*Cd86*)	Mm00444543_m1	NM_019388.3
**Genes**	**Forward Primer**	**Reverse Primer**
*CD86*	ACGATGGACCCCAGATGCACCA	GCGTCTCCACGGAAACAGCA
*IL1β*	CCTGCAGCTGGAGAGTGTGGAT	TGTGCTCTGCTTGTGAGGTGCT
*CD68*	CCACAGGCAGCACAGTGGACA	TCCACAGCAGAAGCTTTGGCCC
*GAPDH*	TGTGTCCGTCGTGGATCTGA	CCTGCTTCACCACCTTCTTGA
*ARG1*	TTTTAGGGTTACGGCCGGTG	CCTCGAGGCTGTCCTTTTGA

## Supplementary Materials

Supplementary materials can be found at http://www.mdpi.com/1422-0067/19/7/2016/s1.
