# Supplementary material for: Concentrated Conditioned Media from Adipose Tissue Derived Mesenchymal Stem Cells Mitigates Visual Deficits and Retinal Inflammation Following Mild Traumatic Brain Injury
Source: Int J Mol Sci. 2018 Jul 11;19(7):2016. doi: 10.3390/ijms19072016 (PMC6073664; doi:10.3390/ijms19072016)
Supplement: Supplementary file 1 [file ijms-19-02016-s001.zip › ijms-321876-SI.pdf]

Supplemental figure legends

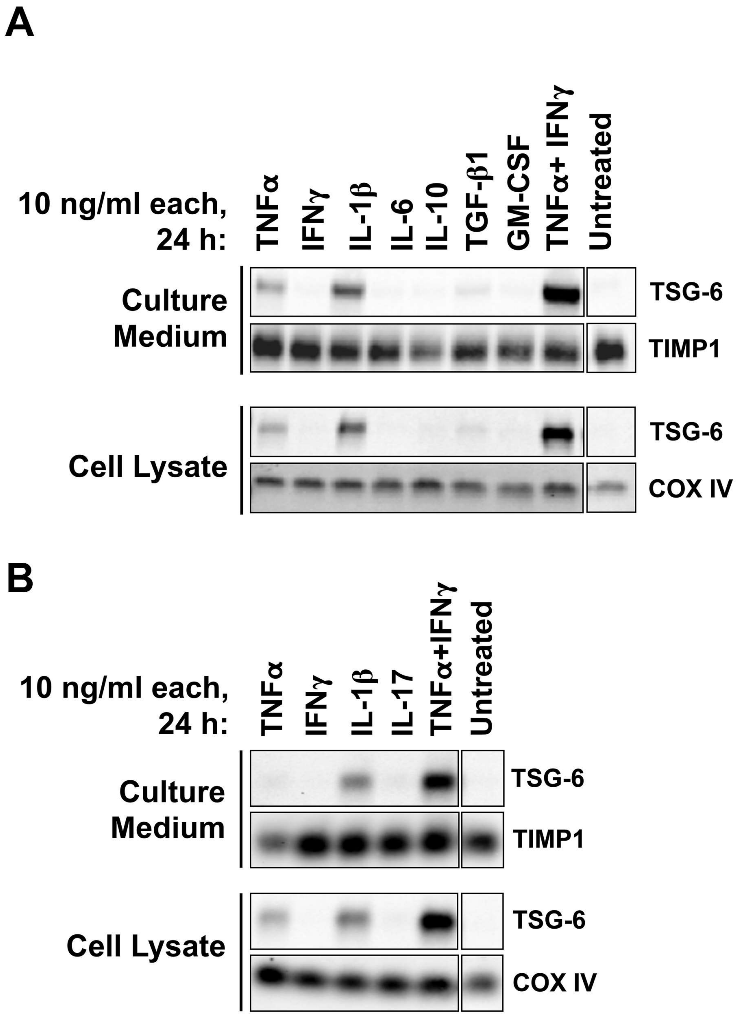

Supplemental Figure S1. TNFα and IFNγ synergize for TSG-6 expression. **(A):** ASCs were cultured in basal medium without or with 10 ng/ml TNFα, IFNγ, IL-1β, IL-6, IL-10, TGF-β1, GM-CSF, or with 10 ng/ml each TNFα and IFNγ for 24 h. Culture medium and cell lysates were immunoblotted for TSG-6 with TIMP1 and COX IV immunoblots serving as loading controls for medium and cell lysates, respectively. **(B):** ASCs were cultured in basal medium without or with 10 ng/ml TNFα, IFNγ, IL-1β, IL-17, or with 10 ng/ml each TNFα and IFNγ for 24 h. Culture medium and cell lysates were immunoblotted for TSG-6 with TIMP1 and COX IV immunoblots serving as loading controls for medium and cell lysates, respectively.

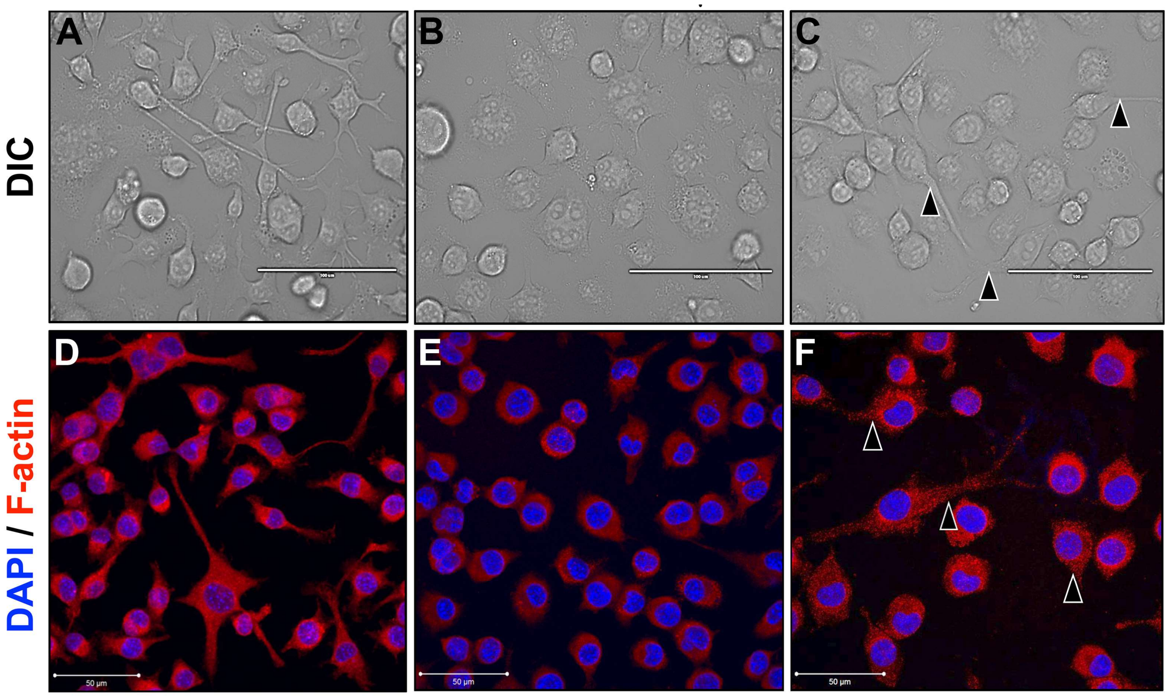

Supplemental Figure S2. ASC-CCM treatment preserves microglial morphology after the LPS (100 ng/ml) and IFNγ (10 ng/ml) exposure as shown by Differential interference contrast (DIC) microscopy (A-C) and F-actin stained confocal micrographs (D-F). **(A&D):** In unstimulated conditions, processes are well maintained while processes are reduced after stimulation with LPS and IFNγ combination **(B&E)** and preserved by ASC-CCM pre-incubation (**C&F**, arrowheads). Scale bars = 100μm (A-C) and 50μm (D-F). Data represent a single experiment performed in duplicates and repeated with similar results with other donor derived ASC-CCM.

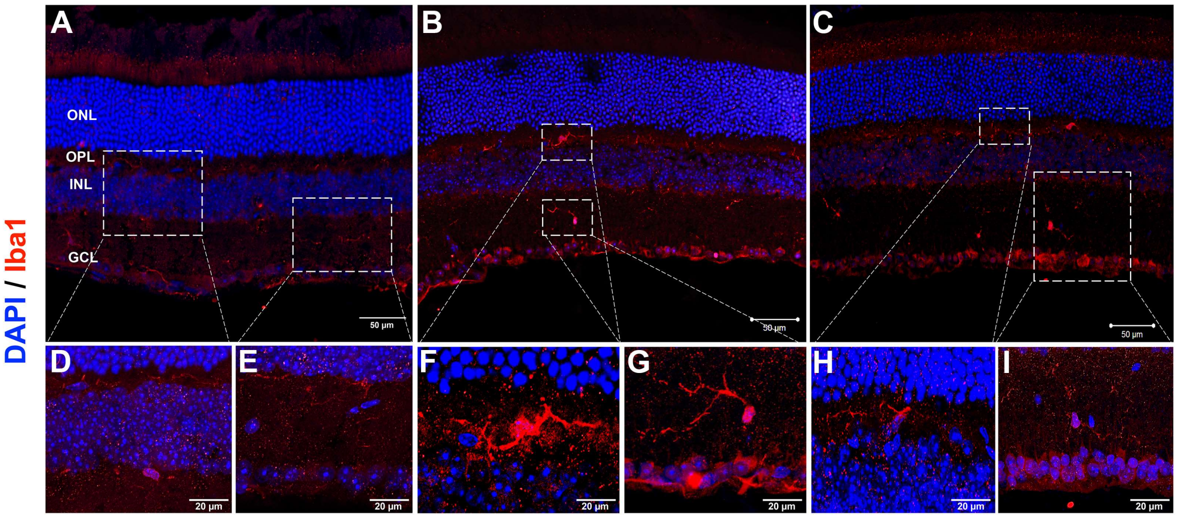

Supplemental Figure S3. ASC-CCM reduces blast-induced alteration of microglia in blast injury mice. Merged confocal micrographs showing morphological alteration in retina by microglial expression marker ionized calcium binding adapter molecule 1 (Iba1) in three different experimental conditions in the left eye. **(A):** Microglia of sham group retinas are ramified with thin processes (higher magnification, D-E: white dotted box). **(B):** Microglia of blast exposed retina are amoeboid in shape, with swollen soma and thick processes indicating hypertrophic changes (higher magnification, F-G: white dotted box). **(C):** Microglia of ASC-CCM group retinas are ramified with thin processes (higher magnification, H-I: white dotted box). All images are z-stacked merged images with higher magnifications (63X) from sham, blast and blast with ASC-CCM injected retina from corresponding position (indicated with dotted box). Data represents 4 animals/group. Scale bars=50μm (A-C) and 20μm (D-I).

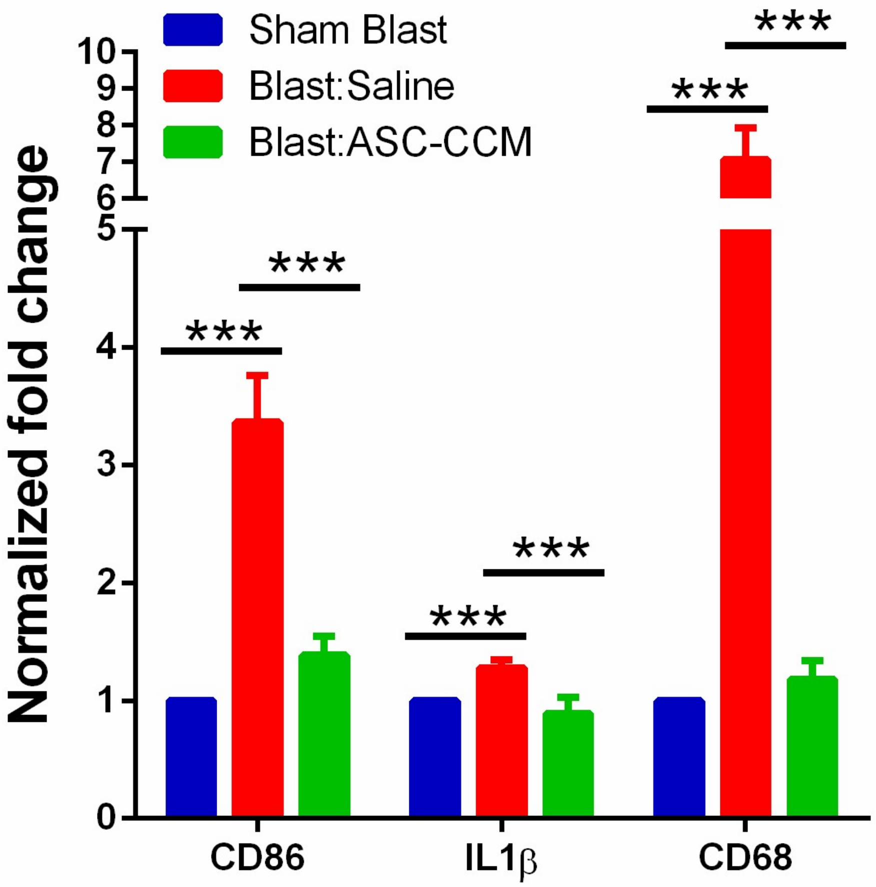

Supplemental Figure S4. ASC-CCM reduces acute retinal inflammation in blast injury mice. Blast mice 3 days post blast and intravitreal injection of saline or ASC-CCM were used in the assessment of gene expression by Taqman qPCR, with results expressed as fold change normalized to internal control (18s rRNA) in the study groups. Data represent Mean ± SEM from n=5-8 animals/group performed in duplicates. ***, $p<0.001$.
